# Production and Incorporation of Calcium-Hydrolyzed Nanoparticles in Alkali-Activated Mine Tailings

**DOI:** 10.3390/nano13121875

**Published:** 2023-06-17

**Authors:** Yibran Perera-Mercado, Nan Zhang, Ahmadreza Hedayat, Linda Figueroa, Esmeralda Saucedo-Salazar, Cara Clements, Héctor Gelber Bolaños Sosa, Néstor Tupa, Isaac Yanqui Morales, Reynaldo Sabino Canahua Loza

**Affiliations:** 1 Department of Civil and Environmental Engineering, Colorado School of Mines, 1500 Illinois St., Golden, CO 8040, USA; zhang_nan@sdu.edu.cn (N.Z.); hedayat@mines.edu (A.H.); lfiguero@mines.edu (L.F.); caraclements@mines.edu (C.C.); 2 Central Laboratory for Analytical Instrumentation, Research Center for Applied Chemistry (CIQA), Blvd. Enrique Reyna Hermosillo 140, Saltillo C.P. 25294, Coahuila, Mexico; esmeralda.saucedo@ciqa.edu.mx; 3 Department of Metallurgical and Environmental Engineering, Faculty of Process Engineering, Universidad Nacional de San Agustín de Arequipa, Santa Catalina Nro. 117, Arequipa, Arequipa C.P. 04001, Peru; hbolanos@unsa.edu.pe (H.G.B.S.); rcanahua@unsa.edu.pe (R.S.C.L.); 4 Department of Civil Engineering, Faculty of Civil Engineering, Universidad Nacional de San Agustín de Arequipa, Santa Catalina Nro. 117, Arequipa, Arequipa C.P. 04001, Peru; ntupaf@unsa.edu.pe (N.T.); iyanqui@unsa.edu.pe (I.Y.M.)

**Keywords:** sol–gel, surfactant, nanoparticles, calcium hydroxide, nano-solutions, alkali-activated materials

## Abstract

This work presented the production and incorporation of calcium-hydrolyzed nano-solutions at three concentrations (1, 2, and 3 wt.%) in alkali-activated gold mine tailings (MTs) from Arequipa, Perú. As the primary activator solution, a sodium hydroxide (NaOH) solution at 10 M was used. Calcium-hydrolyzed nanoparticles with a particle size of 10 nm were localized inside self-assembled molecular spherical systems (micelles) with diameters of less than 80 nm that were well-dispersed in aqueous solutions and acted as secondary activator, and also as additional calcium resource for alkali-activated materials (AAMs) based on low-calcium gold MTs. High-resolution transmission electron microscopy/energy-dispersive X-ray spectroscopy (HR-TEM/EDS) analyses were carried out to characterize the morphology, size, and structure of the calcium-hydrolyzed nanoparticles. Fourier transform infrared (FTIR) analyses were then used to understand the chemical bonding interactions in the calcium-hydrolyzed nanoparticles and in the AAMs. Scanning electron microscopy/energy-dispersive X-ray spectroscopy (SEM/EDS) and quantitative X-ray diffraction (QXRD) were performed to study the structural, chemical, and phase compositions of the AAMs; uniaxial compressive tests evaluated the compressive strength of the reaction AAMs; and nitrogen adsorption–desorption analyses measured porosity changes in the AAMs at the nanostructure level. The results indicated that the main cementing product generated was amorphous binder gel with low quantities of nanostructured C-S-H and C-A-S-H phases. The surplus production of this amorphous binder gel produced denser AAMs at the micro-level and nano-level (macroporous systems). In addition, each increase in the concentration of calcium-hydrolyzed nano-solution had a direct/proportional effect on the mechanical properties of the AAM samples. AAM with 3 wt.% calcium-hydrolyzed nano-solution had the highest compressive strength, with a value of 15.16 MPa, which represented an increase of 62% compared with the original system without nanoparticles that were aged under the same conditions at 70 °C for seven days. These results provided useful information about the positive effect of calcium-hydrolyzed nanoparticles on gold MTs and their conversion into sustainable building materials through alkali activation.

## 1. Introduction

The mining industry generates significant quantities of mine tailings (MTs), with an estimated total annual amount of over 7 billion tons globally [1]. The option of repurposing mine tailings in the form of building materials is very attractive and yet relatively unexplored. Furthermore, alternative eco-efficient binders for producing construction composites primarily developed from industrial waste, such as MTs, are promising for addressing global solid waste management goals. In this respect, alkali-activated materials (AAMs) and geopolymer-based systems are considered alternative potential candidates to ordinary Portland cement systems [2,3].

According to Provis [4], alkali activation is the generic term that is applied to the reaction of solid aluminosilicate (precursor) under alkaline conditions (activator) to produce a hardened binder that is based on a combination of hydrous alkali–aluminosilicate and/or alkali–alkali, earth–aluminosilicate phases [4]. Therefore, AAMs can be produced by mixing silicon- and aluminum-rich raw materials with a highly alkaline solution. Silicon (Si) and aluminum (Al) are released into the solution under high-pH conditions and start to form a new three-dimensional concrete-like hardened structure with high mechanical and chemical performance. In addition to Si and Al, elements such as *x* = Fe, Na, K, Mg, or Ca can react in matrix formation, producing additional/other hydrate products ((*x*)-A-S-H) that can affect the final properties of the structure in a positive way [1], e.g., by densifying the microstructure, reducing porosity and permeability, and making the final microstructure more homogeneous, which guarantees the mechanical strength of the final products [5,6,7,8,9,10]. In addition, the widely used term geopolymers refers, in fact, to a subclass of AAMs (although a consensus was not yet reached on the terminology). Geopolymers are highly coordinated binder systems that have low calcium content and whose network almost exclusively consists of aluminosilicate. The network comprises SiO_4_ and AlO_4_ tetrahedra that are linked by sharing oxygen atoms [11,12,13].

On the other hand, nanomaterials applied to cement-based materials are having an incredible impact on the construction field, where a huge variety of materials, including nano-silica (SiO_2_) [14,15,16,17,18], nano-iron oxide (Fe_2_O_3_) [19,20,21], nano-titanium oxide (TiO_2_) [22,23,24], nano-alumina (Al_2_O_3_) [25,26], nano-calcite (CaCO_3_) [6,27,28], nanotubes [29,30], and nano-clay particles [26,31], were developed/applied in diverse cementitious systems. Therefore, the use of nanoparticles instead of microparticulate additives in cementing systems [32] brings advantages to the construction field, including the following: (1) The production and assembly of materials in the nanometric scale range offers the possibility of the development of new cement additives, such as novel superplasticizers, nanoparticles, or nano-reinforcements [5,33]. (2) The use of nanoparticles provides the capacity to chemically/physically modify the final structures and properties of cementitious materials. (3) Due to their ultrafine size, nanoparticles possess unique physical and chemical properties that are not found in conventional materials and are, thus, being applied in many fields to fabricate new materials with novel functions [34]. (4) Relatively small quantities of nanosized materials are sufficient to improve the performance of composites [35]. In general, the modification of materials with nanotechnology guarantees the optimization of material behavior, as well as the performance that is needed to improve the mechanical properties, porosity change characteristics, durability performance, and sustainability of the final building materials [32].

There are several physical and chemical technologies involved in producing nanoparticles. However, numerous researchers prefer sol–gel technology over other methods to control materials at the atomic scale due to the versatility and high precision of sol–gel technology in the generation of final products. The sol–gel process involves the conversion of monomers into a colloidal solution (sol), which acts as the precursor to an integrated network (or gel) of either discrete particles or networks [31].

In the last few decades, several researchers modified the sol–gel method to produce nanoparticles by using surface-active substances, also known as surfactants, which preferentially adsorb at air–liquid, liquid–liquid, or liquid–solid interfaces. Surfactants are generally classified in two types: (1) ionic surfactants and (2) non-ionic surfactants [36]. One of the most used surfactants to produce calcium-based nanoparticles [37,38,39,40,41] is non-ionic Triton X-100 surfactant (C_14_H_22_O(C_2_H_4_O)_10_), which is composed of a neutral head group, often compiled by a hydroxyl group, and a hydrophobic body. Triton X-100 is adsorbed on the surface of primary Ca(OH)_2_ nanoparticles due to the hydrogen bonds between the hydroxyl group (OH^−^) and the –O of the polyoxyethylene group of Triton X-100, forming a steric barrier [42]. Therefore, the surfactant micelles formed in aqueous systems can encapsulate the calcium species and maintain well-dispersed particles in the sol production step of the sol–gel process. The micelles can prevent the gelation step by restricting the growth, agglomeration, and/or precipitation of the particles using particle-size control under specific experimental conditions, such as concentration, pH, temperature, and stirring rate. Thus, the sol–gel method modified using surfactants is a good technique for producing well-dispersed calcium nano-systems that can be used in the AAM field.

On the other hand, Ca(OH)_2_ was used as a common activator of aluminosilicates such as blast furnace slag and fly ash and was also applied to materials such as metakaolin [43,44]. An advantage of this activator is its low cost compared with compounds based on sodium and potassium. On the global market, the cost of calcium hydroxide is at least five to six times lower than the cost of sodium hydroxide [43]. Therefore, diverse researchers investigated the use/application of different types of calcium nanoparticles in cementitious systems to generate physicochemical alterations in their final structures, where some of the most interesting results included the production of additional calcium silicate hydrate (C-S-H) and calcium aluminosilicate hydrate (C-A-S-H) gels with a direct impact on the properties of the final cementitious systems [6,7,8]. It is important to mention that the nanostructure of cementitious gels, e.g., alkali-activated aluminosilicate products, cannot be readily characterized due to their amorphous nature and the complex multiphase system generated due to their chemical composition [45]. Regularly, AAM/geopolymer binder phases are often assumed to be simply formed by the hardening of amorphous aluminosilicate gels, with no description nor analysis of any potential crystallization pathway [46]. However, several authors explored the structure of gels formed during the alkaline activation of aluminosilicate raw minerals and their main reaction products, which, in some cases, show semi-crystalline order [46,47].

Kiventerä et al. [48] reported that gold MTs have low reactivity and require the addition of a co-binder to achieve a strong matrix via alkali activation, but this is not an effective method to stabilize poorly reactive gold MTs with low calcium content in their chemical composition; hence, other approaches are needed. The goal of the present study was to investigate the effect of calcium-hydrolyzed nano-solutions on the strength and structure of alkali-activated mine tailings. The specific objectives of this study were to (a) synthesize and characterize stable nano-solutions rich in calcium-hydrolyzed species for use as calcium resource additives and as additional alkaline solutions for gold MT paste formulations, (b) investigate the potential of alkaline calcium nano-solutions for modifying the final physicochemical properties of AAMs, and (c) provide evidence based on AAM specimen evaluations to understand the generation of the additional cementing binders capable of densifying AAMs at the nanostructure level due to their role in reducing porosity, which is essential to extending the performance of AAM systems.

## 2. Materials and Methods

### 2.1. Synthesis of Calcium-Hydrolyzed Nano-Solutions

**Figure 1:**
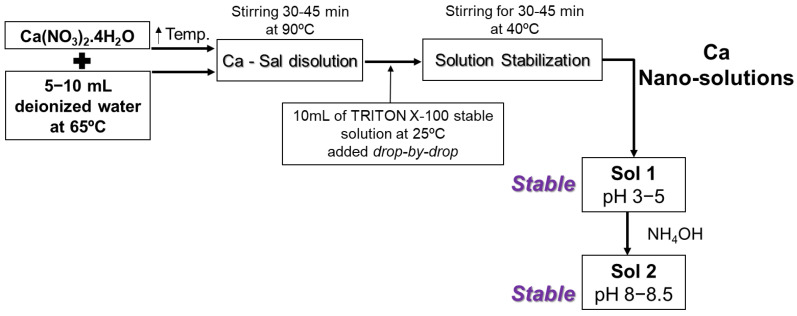
Calcium-hydrolyzed nano-solution synthesis procedure.

In this study, calcium nitrate tetrahydrate (Ca(NO_3_)_2_·4H_2_O) at 98% (extra pure; Acros Organics, NJ, USA), ammonium hydroxide (NH_4_OH) at 28–30% (VWR Chemicals, Radnor, PA, USA), and Triton^®^ X-100 BP151-100 electrophoresis were used for the synthesis of calcium-hydrolyzed nano-solutions. Various previous studies addressed the synthesis and characterization of calcium hydroxide nanoparticles obtained from salt solutions using sol–gel; most of these studies were concerned with hydroxides and the effects of temperature, pH, ionic concentration, and the type of surfactant. High temperature was shown to be necessary to obtain very fine particles, while the properties of the inorganic materials synthesized strongly depend on the conditions of the environment in which they are generated [49]. In this study, calcium nitrate tetrahydrate was dissolved in deionized water at a solution temperature of 90 °C and was stirred for 30 to 45 min. Simultaneously, a 10 mL solution of Triton X-100 surfactant was prepared using the double value of the Triton X-100 critical micelle concentration (CMC) to prevent surfactant losses in the container transfer stages and ensure reaching at least the CMC required for the generation of spherical micelles; the solution was slowly stirred at 25 °C for 30 min. Then, the surfactant solution was added drop by drop to the previous calcium solution. This new mixture was allowed to stabilize under continuous stirring for 30 to 45 min until the solution temperature reached 40 °C. Depending on the concentration of calcium nitrate tetrahydrate in the solution, the pH of this first sol, “Sol 1”, was between 3 and 5. Finally, a few drops of diluted NH_4_OH solution were added under continuous stirring to “Sol 1” until it reached a final pH value between 8 and 8.5; this final solution was called “Sol 2” (Figure 1).

### 2.2. Alkaline Activation Process of MT Samples—Cubic Sample Preparation

Prior to mixing, raw MTs were manually crushed and sieved to control the particle size with 30-mesh filtering (particle size below 600 µm). Then, primary NaOH solutions at 10 M were prepared by mixing water with NaOH pellets at 97% purity (Sigma-Aldrich, Burlington, MA, USA) according to a previous study by Zhang et al. [50]; the solutions were then placed at room temperature (21 ± 1 °C) for 30 min to dissipate the heat generated during dissolution. AAM pastes were then made by hand by combining the MTs with 10 M NaOH solution until a uniform paste was obtained. Then, secondary solutions of well-dispersed calcium-hydrolyzed nanoparticles (at the three different concentrations of 1, 2, and 3 wt.%) were atomized on the pastes and mixed well by hand for 10 min to ensure a uniform final paste mixture. The water/mass ratios used for the original AAM and the AAM samples with calcium nanoparticles were 0.18 and 0.17, respectively.

The pastes were placed in cubic molds and compacted into three different layers using a Harvard miniature compaction (HMC) tamper. Each layer was tamped 71 times, which was calculated as equivalent to the HMC mold such that the number per unit cross-sectional area was constant. WD-40 lubricant was used to release the samples from the molds. The cubic samples were demolded, put in containers, and covered with plastic wrapping to avoid water evaporation in the curing step and then placed in an oven at a slightly elevated temperature of 40 ± 1 °C for 24 h. After that, the oven temperature was increased to 70 ± 1 °C for three days. After four days of curing, the plastic wrapping was removed, and the specimens were placed in the oven for three more days for drying at the same temperature of 70 ± 1 °C. A total of three cubic specimens for each system in the study were cast not only to test the compressive behavior (Section 2.4) but also for the entire characterization of all these systems.

### 2.3. High-Resolution Transmission Electron Microscopy (HR-TEM) Characterization

A drop of the concentrated calcium-hydrolyzed nano-solutions was diluted in distilled water; then, a small drop of the well-diluted nano-solutions was characterized with high-resolution transmission electron microscopy (HR-TEM), using an FEI Titan 80–300 microscope capable of generating selected-area electron diffraction (SAED) patterns, and by performing qualitative chemical analysis with energy-dispersive spectroscopy (EDS) of X-rays. In addition, fast Fourier transform (FFT) analyses were carried out on specific areas of the synthetized nanostructures for atomic plane identification. These analyses were performed to confirm the shape, size, and chemical composition of the nanoparticles and their structural phases in order to identify the presence of Ca(OH)_2_ nanocrystalline particles.

### 2.4. Uniaxial Compressive Tests

The compressive strength of the reaction MT samples was tested following ASTM C109 [51]. An MTS Landmark 370.10 machine at a constant displacement rate of 0.21 mm/min was used to evaluate the different AAM systems. The cubic samples cured for seven days were first placed between two metallic plates to ensure that the surfaces were sufficiently flat for the compression test. Under these given experimental conditions, a total of three cubes/measurements were taken and averaged.

Samples from the center of the broken cubes evaluated in this section were taken and manually crushed in an Agate mortar until they became very-well-dispersed powders; powder of each sample evaluated in this research was used in the following characterization steps.

### 2.5. Fourier Transform Infrared (FTIR) Characterization

A Thermo Electron Nicolet 4700 Fourier transform infrared (FTIR) spectrometer was used to collect spectra in transmittance mode at specific frequencies ranging from 4000 to 400 cm^−1^. The FTIR system was equipped with a KBr detector. The samples (powders) were placed on the FTIR collector window under room environment conditions. The resulting spectrum analyses were interpreted to identify the specific functional groups in the analyzed samples.

### 2.6. Quantitative X-ray Diffraction (QXRD) Characterization

XRD analysis was performed to investigate the phase composition of the MT and AAM systems. Measurements were made using an X’Pert PRO MPD X-ray diffraction system. The X-ray radiation was Cu Kα, λ = 1.5418 Å, and the current was 40 mA with a tension of 45 kV. The scan time and instrument parameters were identical for all the samples, with a 2θ angle range between 10° and 90°. To perform QXRD analysis on the AAM specimens, X’Pert HighScore software 5.1, which includes the Rietveld method [52,53], was used. The internal standard material added was 9.1 wt.% synthetic diamond with particle sizes between 2 and 4 μm [54,55]. The samples were prepared by intergrading each one of them with synthetic diamond using a porcelain mortar. Finally, the powder diffraction file (PDF) codes (International Centre for Diffraction Data—ICDD) were used to identify the crystalline phases.

### 2.7. Scanning Electron Microscopy/Energy-Dispersive Spectroscopy (SEM/EDS) Characterization

After the uniaxial compression test, the AAM samples (powders) were characterized using an FEI QUANTA 600i Environmental scanning electron microscope, which used a tungsten filament and was equipped with an EDAX Element SDD EDS detector. The semiquantitative chemical composition was reported by averaging ten measurements for each specimen. The morphology of the fracture surface of each sample, which was specifically not polished, was determined.

### 2.8. Nitrogen Adsorption–Desorption Analyses

Nitrogen adsorption analyses were performed using a Micromeritics ASAP 2020 Plus machine. Before the experiments, the AAM samples (powders) were degassed under vacuum at 70 °C for 24 h. The pore-size distribution was calculated using the Barrett, Joyner, and Halenda (BJH) method based on the desorption branches of nitrogen isotherms. In addition, Halsey–Faas correction was used; this procedure gave the dV/dD incremental pore volume (units of cm^3^/g/A) for each sample evaluated. The specific surface areas were calculated using the Brunauer–Emmett–Teller (BET) theory of single-layer adsorption for all samples studied.

## 3. Results and Discussion

### 3.1. Raw Material Characterization

Gold MT samples from Vitor (Arequipa, Perú) were used in this study. The geotechnical characterizations of these MTs were reported in previous works [56,57], and a summary of their characteristics is listed as follows: (1) mean particle size D_50_ = 0.086 mm with fine percentage equal to 41.16%; (2) coefficient of uniformity Cu = 5.05. The MTs were classified as silty sand (SS) with low plasticity according to USCS classification standards, i.e., low liquid limit (23.08%), low plasticity (PI = 1.34%), and low capacity for holding water (A = 0.033) [56,57].

**Figure 2:**
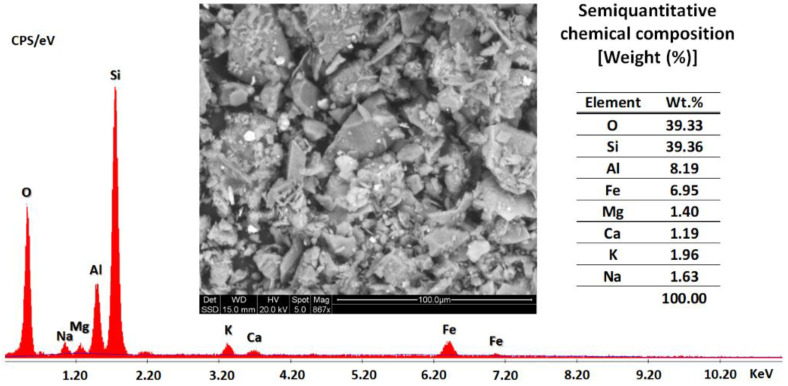
SEM and EDS semiquantitative chemical analyses of gold mine tailings (Vitor).

SEM and EDS semiquantitative chemical composition analyses of the gold mine tailings (Vitor) are shown in Figure 2. The mine tailing SEM micrograph exhibited the typical microstructure of particles with different angularity and finer particles attached to the surface of coarse particles. The EDS semiquantitative chemical composition indicated silicon (Si) as the primary element of the composition of the studied gold mine tailings, where aluminum (Al) and iron (Fe) were the majority secondary elements, and magnesium (Mg), calcium (Ca), potassium (K), and sodium (Na) were present in minor quantities.

The quantitative X-ray diffraction data of these gold MTs were also reported in a previous research work [57]; the results indicate that the main crystalline mineral phase was quartz (SiO_2_) [50,58], followed by muscovite (KAl_2_(FOH)_2_ or (KF)_2_(Al_2_O_3_)_3_(-SiO_2_)_6_) [50], magnetite (Fe_3_O_4_) [41,59], albite (Na(AlSi_3_O_8_)) [50,58,60], C-A-S-H (Ca_3_Al(Al_3_SiO_10_)(OH)_2_) [57], and N-A-S-H (Na_17.6_(Al_16_Si_56_O_144_)(H_2_O)_38.4_) [57]. Additionally, a low percentage of amorphous phase was also identified [57].

### 3.2. Synthesis of Calcium-Hydrolyzed Nano-Solutions

The nano-solution synthesis methodology discussed in Section 2.1. focused on the production of well-dispersed calcium-hydrolyzed nanoparticles in suitable alkaline aqueous solutions that is compatible with the gold MT alkaline activation process using NaOH solution as the activator. Therefore, the effects of the synthesis parameters (e.g., temperature, pH, and concentration) were analyzed in detail and are explained in the next sections. For example, the experimental procedure shown in Figure 1 revealed that the temperature of the reaction for calcium solutions must be equal to or higher than 60 °C; otherwise, it is not possible to obtain water molecular separation from Ca(NO_3_)_2_·4H_2_O according to the following chemical reaction (Equation (1)):

(1) $$\mathrm{Ca}{\left({\mathrm{NO}}_{3}\right)}_{2}\cdot 4H_{2}O\;\overset{\Delta }{\to }\;\mathrm{Ca}{\left({\mathrm{NO}}_{3}\right)}_{2}+4H_{2}O\;\left(60-100\;℃\right)$$

After the molecular decomposition of the Ca precursor, the hydrolysis step of the calcium species started as the pH and the solution temperature were changed. Finally, stable transparent calcium nano-solution was obtained at pH 8.5, and the product was characterized using different methods.

#### 3.2.1. FTIR of Calcium-Hydrolyzed Nano-Solutions

**Figure 3:**
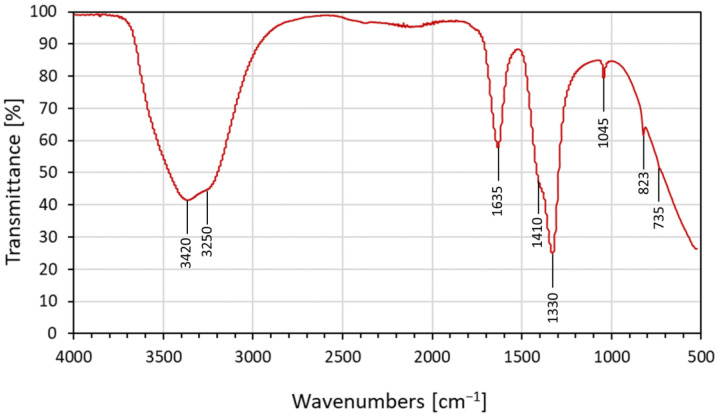
FTIR spectrum of calcium-hydrolyzed nano-solution at pH = 8.5.

The FTIR spectrum in Figure 3 was obtained from transparent nano-solution at pH = 8.5, where the calcium species were still in a hydrolyzed state in the solution and were confined into the surfactant micellar system. The data presented below refer to the ionic interactions between hydrated Ca^+^ and NO_3_^−^ ions in solution [61].

The FTIR spectrum shows the characteristic peaks of water at 3250–3420 cm^−1^, as well as 1635 cm^−1^, due to hydroxyl (O–H) stretching and bending modes, respectively [62,63]. The presence of the O–H group was caused by water from the calcium-hydrolyzed aqueous nano-solutions [62]. During the process of calcium-hydrolyzed nano-solution synthesis, ammonium nitrate (NH_4_NO_3_) was generated as a byproduct, and it remained dissolved into the aqueous medium according to the following reaction (Equation (2)):

(2) $$\mathrm{Ca}{\left({\mathrm{NO}}_{3}\right)}_{2}\cdot 4H_{2}O+2{\mathrm{NH}}_{4}\mathrm{OH}\;\to \mathrm{Ca}{\left(\mathrm{OH}\right)}_{2}+2{\mathrm{NH}}_{4}{\mathrm{NO}}_{3}+4H_{2}O\;$$

The signals between 1300 and 1500 cm^−1^ corresponded to the asymmetric deformation mode of NH_4_^+^. It was also possible to determine two simultaneous vibrations associated with asymmetric stretching and possible in-plane deformation modes of NO_3_**^−^** species. In addition, a totally symmetric stretching mode of NO_3_**^−^** was identified at 1045 cm^−1^; at 823 cm^−1^, it was possible to identify the out-of-plane deformation mode of NO_3_**^−^** [61,64]. The low-intensity signal at 735 cm^−1^ supports the theory of ionic interaction, which, according to Irish et al. [61], was due to the perturbation between one calcium ion and one nitrate ion; this corroborated the simultaneous presence of both species in the nano-solutions at pH = 8.5. Therefore, the calcium-hydrated nano-solution at pH 8.5 contained as the principal compound a mixture of calcium-hydrolyzed species with a very low population of crystalline Ca(OH)_2_ nanoparticles, which are normally obtained in higher-alkaline-pH solutions [37]. At pH values equal to or higher than 12, Ca(OH)_2_ precipitated due to an increase in the particle size of the calcium-hydrated species in the hydrolysis step, which is a non-desirable effect in the current research work. Thus, a balance of diverse calcium species in water, such as Ca^2+^, Ca(OH)^+^, and Ca(OH)_2_ [65], was expected. Additionally, in concentrated calcium nano-solutions, the solvated nitrate ion shares water molecules with the solvated calcium ions, which could generate a potential interaction among calcium–H_2_O–nitrate species [61].

#### 3.2.2. HR-TEM of Calcium-Hydrolyzed Nano-Solutions

**Figure 4:**
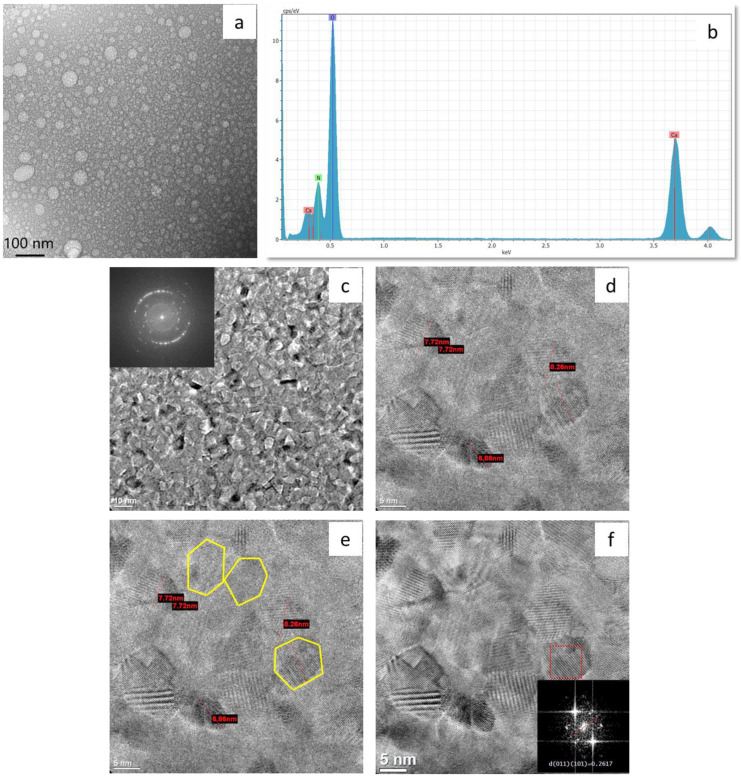
HR-TEM micrographs of calcium-hydrolyzed nano-solution at pH = 8.5: (**a**) general view of the systems formed by well-dispersed spherical particles and (**b**) their qualitative chemical EDS analysis; (**c**) detail of the internal structure of the spherical particles shown in (**a**) with SAED pattern; (**d**) measurement of the hexagonal-like particles sizes, highlighted in yellow (**e**); (**f**) FFT image of a single-hexagonal particle as identified in (**d**,**e**).

The HR-TEM results are shown in Figure 4 with their corresponding SAED patterns, their elemental–chemical EDS analysis, and the FFT image obtained to distinguish the crystalline structure of the nanoparticles observed in calcium-hydrolyzed nano-solution at pH 8.5. These analyses were performed to confirm the shape, size, and chemical composition of the nanoparticles and their structural phases in order to identify the presence of Ca(OH)_2_ nanocrystalline particles to corroborate the FTIR results.

Figure 4a shows the distribution of spherical particles with a variant particle-size distribution. The diameters of the particles were shorter than 80 nm. The spherical morphologies indicate the effect of non-ionic surfactant when it reached the CMC, which made it possible to achieve both micelle formation, to provide a well-dispersed system of particles in aqueous solution, and direct control over the growing of Ca(OH)_2_ crystals by encapsulating the calcium-hydrolyzed species. Well-dispersed nanoparticle production based on a self-assembled molecular cluster system (micelles) in aqueous solutions was previously reported by several researchers [38,39,65]. Furthermore, Paradles [40] indicated that under controlled experimental parameter conditions, non-ionic Triton X-100 surfactant produced micelles with a radius of 5.1 nm in aqueous solution. However, it was expected that the micelle radius could change due to the effect of different conditions, such as pH, temperature, chemical species concentration, stirring rates, and type of solvent, among others. One of the factors that could have affected the micelle size in this study was the change in solution temperature during the synthesis process. During calcium-hydrolyzed nano-solution production, the temperature was changed from room temperature to 90 °C when the surfactant solution was incorporated drop by drop under constant stirring. This abrupt temperature change may have increased the radius of the micelles, in accordance with Paradles’ results [40].

On the other hand, it could be seen that the micelle spherical particles were filled with smaller particles. Figure 4c shows a higher magnification of the micelle internal structure that corroborates the presence of nanoparticles with very homogenous particle sizes that were less than 10 nm. Bright electron diffraction spots can be seen in the SAED diffraction pattern, indicating that the internal structure of the micelle spherical particles was formed by polycrystalline nanoparticles with chemical composition of calcium (Ca), oxygen (O), and nitrogen (N), as shown in the EDS spectra in Figure 4b. The nitrogen (N) signal was expected due to the calcium salt precursor used in the calcium-hydrolyzed nano-solution synthesis step.

Figure 4d, which is a magnification of Figure 4c, shows that some of the hexagonal particles developed well-defined edges, which illustrates the crystal growth pattern common to Ca(OH)_2_. In addition, some particles showed ends that were fragmented or incomplete because the nano-solutions did not reach the high pH value (≈12) at which all Ca(OH)_2_ particles were expected to complete their hydrolysis step, which made their growth and precipitation possible.

Separated individual Ca(OH)_2_ tetrahedral crystals with particle sizes smaller than 10 nm are clearly highlighted in Figure 4e. These smaller particles with the higher hexagonal symmetry of Ca(OH)_2_ crystals were generated when temperatures higher than 60 °C were used during the dissolution of calcium nitrate tetrahydrate in water in the first step of nano-solution production; this was also reported by Ambrosi et al. [37], who used temperatures close to 90 °C during the synthesis process of Ca(OH)_2_ nanoparticles [37]. Additionally, Michalopoulou et al. [42] indicated that the use of non-ionic Triton X-100 during the production of dispersed nano-lime (Ca(OH)_2_) in water resulted in plate-like Ca(OH)_2_ nanoparticles, which are customarily hexagonal, thereby showing the direct effects of smaller size, enhanced width, and more angular shape. The reduced size of the nanoparticles was attributed to the reported interaction between Ca^2+^ and the functional group –CH_2_–CH_2_–O– of Triton X-100, which was proven to inhibit the growth of primary particles and the presence of aggregation phenomena.

In order to verify the crystalline structure inside the hexagonal particles, FFT analysis was performed on the HR-TEM micrograph. Figure 4f shows a very clear hexagonal particle (bottom-left) that was highly ordered, and its FFT image showed well-defined spots. By measuring the interplanar distances (d_hkl_), planes (011) and (101) of portlandite (Ca(OH)_2_) were identified. Based on the XRD pattern, plane (011) had a direct correspondence with the most intense XRD signal at 2θ = 34.11°, and plane (101) belonged to the same plane family, as shown in Table 1. These results corroborate the presence of portlandite (Ca(OH)_2_) crystalline particles in the nano-solutions.

### 3.3. Production and Characterization of AAM Systems

#### 3.3.1. Compressive Strength of AAM Systems

**Figure 5:**
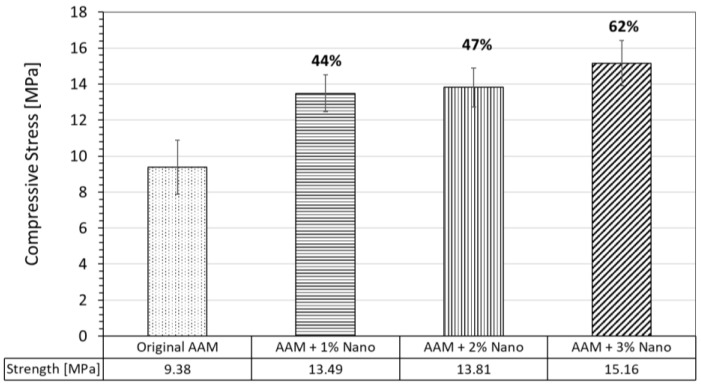
Comparative compressive strength values of the original AAM and AAMs supplemented with calcium-hydrolyzed nano-solutions at three different concentrations.

Uniaxial compression tests were performed on the AAM specimens after seven days of the curing process. The compressive strength average values of all the evaluated systems are reported in Figure 5. It can be seen that the compressive strength of the original AAM cubes was 9.38 MPa. The compressive strength of the AAM samples with calcium-hydrolyzed nano-solutions at three different concentrations (1, 2, and 3 wt.%) of MTs increased for all the samples as follows: the specimens at 1 wt.% achieved the compressive strength of 13.48 MPa, which represents an increase of 44%; for specimens at 2 wt.%, the compressive strength was 13.81 MPa, which corresponds to an increase of 47%; and the specimens at 3 wt.% achieved the compressive strength of 15.16 MPa, which is an increase of 62%.

From a chemical reaction standpoint, when an alkaline activator, such as sodium hydroxide (NaOH) or potassium hydroxide (KOH), is exposed to aluminosilicate minerals, phase dissolution starts, and elements such as aluminum and silicon initiate their hydrolysis processes in the presence of an aqueous medium rich in OH^−^ as follows [66]:

(3) $${SiO}_{2}+2OH^{-}\;\to \;{\left[{SiO}_{2}{\left(OH\right)}_{2}\right]}^{2-}$$

(4) $${\mathrm{Al}}_{2}O_{3}+3H_{2}O+2{\mathrm{OH}}^{-}\;\to \;2{\left[\mathrm{Al}{\left(\mathrm{OH}\right)}_{4}\right]}^{-}$$

The addition of calcium-hydrolyzed nano-solutions to the low-calcium MT systems provided additional chemical reactions based on the pozzolanic properties of calcium-hydrolyzed nanoparticles disperse in aqueous solutions, which simultaneously reacted with the silicon and aluminum hydrolyzed species generated during the alkaline activation process, which affected the physicochemical properties of the final AAMs in a positive way. Some of the additional main products generated in those systems were as follows:

(5) $${\left[{SiO}_{2}{\left(OH\right)}_{2}\right]}^{2-}+Ca{\left[{\left(H_{2}O\right)}_{6}\right]}^{2+}\to C-S-H_{\left(\mathrm{gel}\right)}$$

(6) $$[\mathrm{Al}{\left(\mathrm{OH}\right)}_{4}{]}^{-}+{\left[{\mathrm{SiO}}_{2}{\left(\mathrm{OH}\right)}_{2}\right]}^{2-}+\mathrm{Ca}[{\left(H_{2}O\right)}_{6}{]}^{2+}\to C-A-S-H_{\left(\mathrm{gel}\right)}$$

It is well known that C-S-H gel is able to fill the voids and improve the compactness of different cementing systems, along with the binding matrix, in accordance with Equation (5) [67,68]. In addition, C-S-H gel is also generated during the Portland cement hydration process, and it is the main product responsible for the high-strength development of this cementing system. Furthermore, it is generally believed that C-S-H gel is the component that strengthens the resulting binder [8]. Additionally, the presence of aluminum (Al) in the system allows C-A-S-H gel to be generated, which is important in light of the fact that both gels (C-S-H and C-A-S-H), which are normally non-stoichiometric, are among the hydration products of novel AAMs. Hence, the presence of additional calcium species in the MT alkaline activation step was advantageous because of their role in the formation of hydration products such as C-S-H and C-A-S-H (Equation (6)) gels, which are well-known for the mechanical strength that they impart on cementitious materials [41,68]. Secondary chemical reactions due to the hydrolysis of other elements, such as magnesium, may take place during the inorganic polymerization process, because Mg easily and drastically reacts with water to form Mg(OH)_2_, which, in the presence of a calcium resource, can generate C-M-A-S gel, which can strengthen the microstructure [60]. Chen et al. [9] stated that C-S-H gel may contain Al, Fe, and Mg due to the sorption or co-precipitation processes generated during the hydration process of cementitious materials, where minor components can modify the crystal growth via substitution in the lattice of calcium silicate hydrates. On the other hand, Silva de Vargas et al. [10] reported that the matrix formed by the alkali activation of aluminosilicates using alkaline solution is formed by M-A-S-H gel, where M is an element of the first group of the periodic table (Na^+^ and K^+^ are the most used).

#### 3.3.2. Quantitative X-ray Diffraction (QXRD) Analysis of AAM Systems

Table 2 presents the results of quantitative X-ray diffraction used to identify the mineralogical composition of the original AAM and the AAM samples with added calcium-hydrolyzed nano-solutions. The QXRD data obtained using the Rietveld refinement method (Table 2) show that all AAM samples mainly contained the same crystalline phases as previously identified in the original MTs (Section 3.1) except the N-A-S-H phase. The primary crystalline phases are quartz (SiO_2_) [41,50,69], albite (Na(AlSi_3_O_8_)) [50,58,60], and muscovite (KAl_2_(FOH)_2_ or (KF)_2_(Al_2_O_3_)_3_(-SiO_2_)_6_) [50]. However, some additional crystalline phases were detected in low quantities; these phases are magnetite (Fe_3_O_4_) [41,59], portlandite [Ca(OH)_2_] [41,70,71], calcite (CaCO_3_) [70,72], zeolite [41,72,73,74], C-S-H [70,73,75], and C-A-S-H [76]. The quantification of amorphous phases in each AAM specimen was also determined.

Muscovite appeared to be the main phase capable of releasing higher quantities of silicon and aluminum species during the alkaline dissolution of minerals, followed by the albite and quartz phases, with dissolution rates lower than that of muscovite [77]. Therefore, it was expected that C-S-H and C-A-S-H phases could be generated due to the combination of soluble Ca^2+^ ions from the calcium-hydrolyzed nano-solutions and the silicon and aluminum ions released by the aluminosilicate phases of the MT precursor in the dissolution step in the presence of alkali activator solution [75]. In this case, the main reaction product was an amorphous aluminosilicate cementing phase with low crystallization of C-S-H gel, which took up some percentage of Al in its structure for the generation of additional C-A-S-H gel (Equations (5) and (6)). Although the C-S-H and C-A-S-H phase contents increased as the calcium-hydrolyzed nanoparticle concentration in the AAM systems increased, the low quantity of these phases, as evidenced by the QXRD results, indicates the potential short-range order of the C-S-H and C-A-S-H phases, which is attributed to the possible formation of nanostructured gels.

Additionally, there was a direct increase in amorphous content as the calcium-hydrolyzed nanoparticle concentration increased in the AAM specimens, indicating the production of more cementing binders in the amorphous state [57]. These results suggest that the presence of calcium-hydrolyzed nanoparticles during the MT alkaline activation process positively contributed to the dissolution of the aluminosilicate phases, allowing additional binders to be generated, which ultimately contributed to the strength increase in the AAM specimens (Figure 5). A similar effect was reported by Zhu et al. [78] in the production of alkali-activated metakaolin/slag pastes, where the substitution of metakaolin (with very low calcium content) with slag (with high calcium content) increased the reactivity of the precursors, contributing to a higher reaction rate and accelerating the geopolymerization process. Similarly, Canfield et al. [79] investigated the role of calcium in blended fly ash geopolymers and found that calcium can act as an activator, because calcium species with OH^−^ raise the pH in local environments, which, in turn, increases the dissolution of local aluminosilicate particles and, thus, increases the extent of the inorganic polymeric structure.

On the other hand, the previous low quantity of Ca(OH)_2_ particles identified as part of the calcium-hydrolyzed nano-solutions (Section 3.2.2) could make a direct contribution to the portlandite content of AAM systems. Likewise, an insignificant amount of calcite (CaCO_3_) in the AAM systems was observed, possibly due to the reaction of calcium species with carbon dioxide from air in the presence of an aqueous alkali activator [76,80]. Similarly, the presence of zeolite phase was expected in these types of AAM systems. Provis et al. [46] reported that the identification of nanocrystalline zeolitic materials as a significant component of geopolymeric materials embedded in alkaline amorphous aluminosilicate gel phase is not unexpected. In addition, it is well known that N-A-S-H phase is present in raw MTs (Section 3.1) and that it is a zeolitic precursor [57]. However, it is possible to observe that as calcium-hydrolyzed nanoparticle content increased in the AAM systems, zeolite formation decreased, indicating the potential preference of these systems for generating other phases over zeolite in the presence of calcium species. Finally, the magnetite phase showed a slow dissolution process in the alkali activation step, allowing the slowly released iron species to be part of the formed amorphous cementing binders [9].

#### 3.3.3. FTIR Characterization of AAM Systems

**Figure 6:**
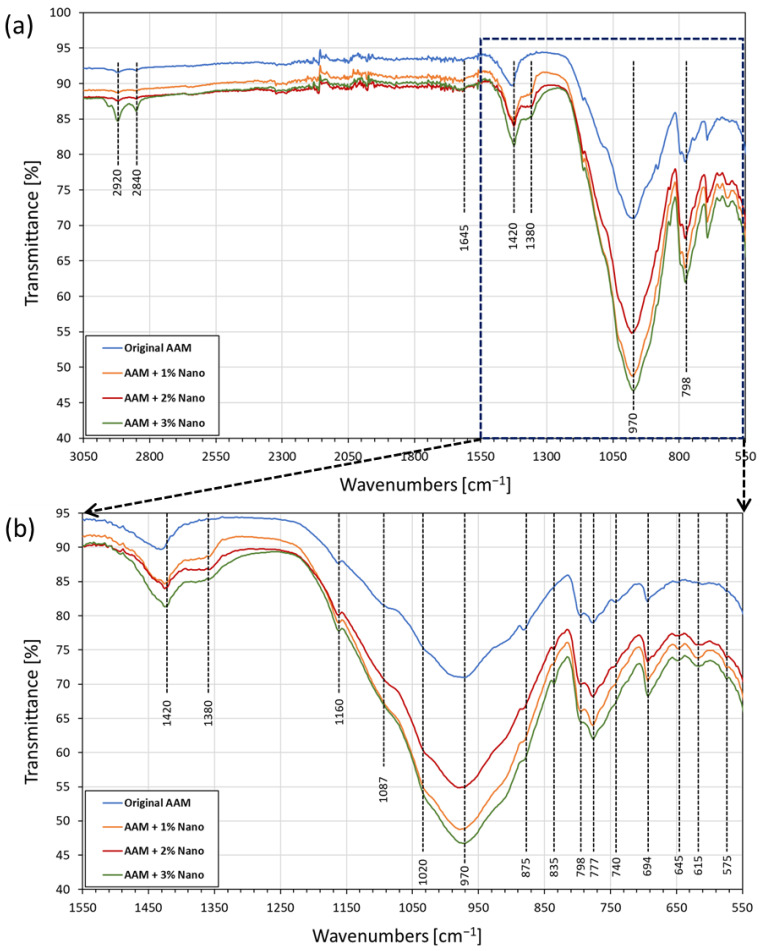
(**a**) FTIR spectra of the original AAM and AAMs supplemented with calcium-hydrated nano-solutions. (**b**) Magnification of zone between 1550 and 550 cm^−1^.

The FTIR spectra of the AAM samples are shown in Figure 6a. The bands observed at 2920 cm^−1^ and 2840 cm^−1^ were attributed to the symmetric and asymmetric stretching of C–H groups, possibly caused by the WD-40 lubricant used in the demolding step of cubic sample preparation [81]; these vibrations were also identified in the original spectra of the Triton X-100 surfactant used in calcium nano-solution synthesis. Figure 6b shows a more detailed view of the FTIR spectra in the region of 1550–550 cm^−1^. The band at about 1420 cm^−1^ was attributed to the stretching vibrations of the C–O bond of carbonates, likely sodium carbonates. Despite the fact that special care was taken to avoid carbonation during the experiments, a small number of carbonates were generated due to the reaction of the activator solution of NaOH with atmospheric CO_2_ [76]. The carbonates within the samples also presented a small protrusion at 875 cm^−1^, which is an out-of-plane bending of the carbonate group. In addition, the signal between 693 and 700 cm^−1^ corresponds to calcium carbonate phase [82] and potentially to the formation of sodium carbonates [83], which corroborates the QXRD results of CaCO_3_ generation in the geopolymerization step.

The small band at about 1380 cm^−1^ was assigned to the nitrates that resulted from the method for the preparation of calcium-hydrolyzed nano-solutions [76]. A moderate intensity band occurred at 835 cm^−1^, possibly corresponding to Si–O bond symmetric stretching vibrations [80]. However, this vibration may have overlapped the signal at 830 cm^−1^ assigned to the bending mode of NO_3_**^−^** [84,85].

On the other hand, there is a clear overlapping of signals in the range of 1200–800 cm^−1^. All the spectra present a huge absorption band in this region, which is typical for aluminosilicate structures and was attributed to the asymmetric stretching vibration of Si–O–Si and Si–O–Al bonds in [SiO_4_]_4_**^−^** and [AlO_4_]_5_**^−^** [76]. In addition, other overlapping signals in this region were attributed to the S–O adsorption band of SO_4_**^−^**^2^, pertinent to the formation of the sulfate-based mineral band around 1020 cm^−1^ [81].

The vibration at 798 cm^−1^ is a characteristic signal of quartz, which is the main mineral compound of the used MTs, and its presence was also corroborated by the XRD patterns [83]. However, the group of signals at 798 cm^−1^, 777 cm^−1^, and 694 cm^−1^ indicates the existence of Si–O–X bonds in the resulting AAM systems (X stands for tetrahedral silicates or aluminates). The magnitude of the absorption bands is related to the amounts of silicates or aluminates and the degree of inorganic polymerization [50]. As the concentration of calcium species increased, there was an increase in the overlapping signal at 740 cm^−1^; apparently, this band is generated by either one or more calcium ions interacting with nitrate ions [86].

According to Kapeluszna et al. [76], the peaks observed in the 800–600 cm^−1^ zone (645 and 615 cm^−1^) were distinguishable in the characteristics structure of C-A-S-H gel vs. Si–O–Si, Al bonds, respectively. In addition, the vibrations between 600 and 400 cm^−1^ (575 cm^−1^) suggest the occurrence of tetrahedrons in silicate and aluminosilicate networks in C-S-H gel and C-A-S-H gel hydrates [76,86,87].

#### 3.3.4. SEM/EDS Characterization of AAM Systems

**Figure 7:**
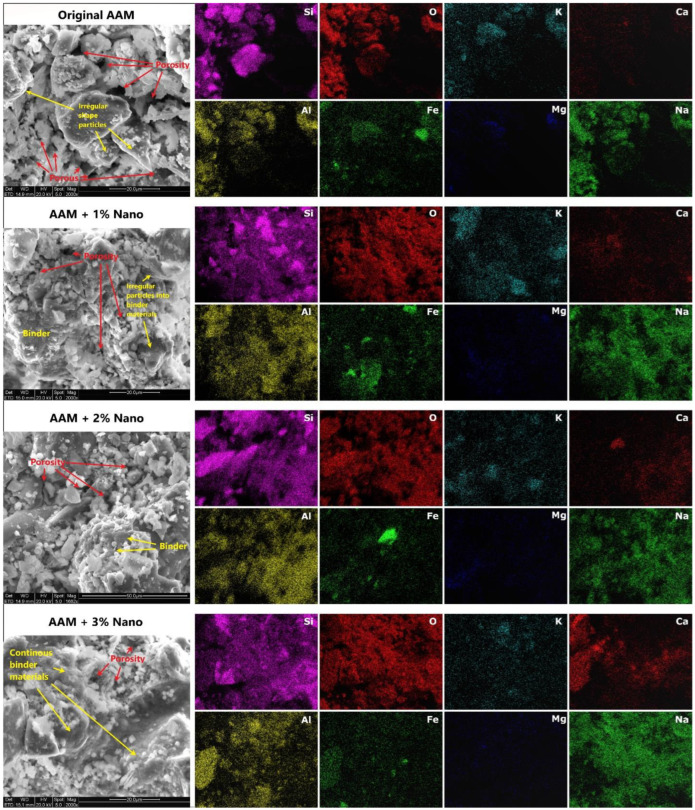
SEM micrographs and EDS elemental mapping analyses of the original AAM and AAMs supplemented with different concentrations of calcium-hydrolyzed nano-solutions.

Figure 7 shows SEM micrographs of the fractured surfaces of the original and calcium-hydrolyzed nano-solution AAMs. These images showed more consolidated structures as the calcium-hydrolyzed nano-solution content increased. It was possible to observe how irregularly shaped particles were covered with cementing binders grown over and between them. Therefore, the densification of the samples was evident as the content of calcium increased in the systems; for instance, the pores observed in the original AAM systems were filled with a continuous phase of cementing products, as clearly observed in the SEM images of the AAM specimen with 3% calcium nanoparticles. Hence, the consolidated microstructure was due to the production of C-S-H and C-A-S-H, and in major quantity, by amorphous cementing binders, which is corroborated by the QXRD results. The chemical compositional maps of the AAM systems indicated several areas with strong signals of Si, Al, O, and Na; furthermore, it was possible to identify secondary elements, such as Fe, K, Mg, and Ca.

In addition, Si, Al, and O dominated the entire mapped area, indicating their abundance, which was expected due to the presence of aluminosilicate minerals in the MT precursor. The comparative EDS mapping of the specific calcium element distribution (Figure 7) showed no agglomerations of calcium nanoparticles, with high dispersion of the latter in the final consolidated specimens. The increase in Ca signals was directly proportional to the content of calcium-hydrolyzed nanoparticles in the systems. Ca, Si, and Al coexisted in the same area, suggesting homogeneous generation of C-S-H, C-A-S-H, and amorphous binder products [88]. Meanwhile, Fe, K, and Mg were also observed in the same regions, as they were part of the original MT precursor. Sodium showed a strong signal in all the AAM samples due to its incorporation into the systems when NaOH was used as alkaline solution activator.

The average of ten EDS measurements of different areas from each AAM specimen is reported in Table 3. These results indicate that the increase in Ca element content in the AAM samples was directly proportional to the increase in calcium-hydrolyzed nano-solutions content.

Based on the SEM images and EDS analyses (Figure 7), the calcium-hydrolyzed nanoparticles had a direct impact on the generation of additional cementing binders, which improved the final consolidated microstructure of the AAM specimens [88]. The reduced porosity at the micro-level suggests that a denser matrix could also contribute to producing stronger final AAM specimens, as was observed in the mechanical properties of the AAMs as the calcium-hydrolyzed nanoparticle concentration increased in each evaluated system.

#### 3.3.5. Nitrogen Adsorption–Desorption Analyses of AAM Systems

**Figure 8:**
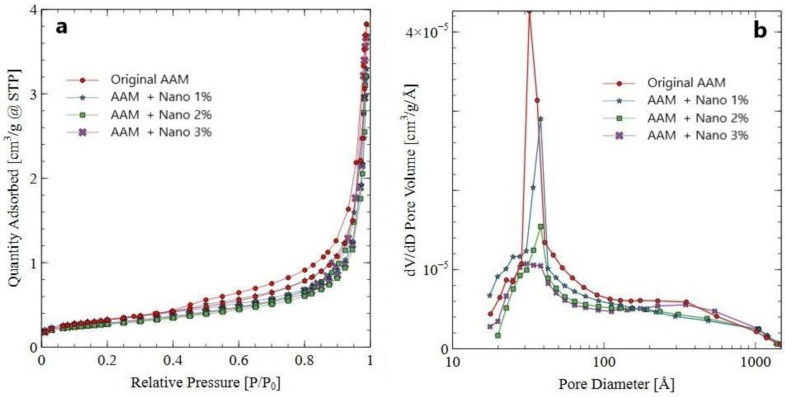
(**a**) Nitrogen adsorption–desorption isotherms and (**b**) pore-size distributions of the original AAM and AAM samples with 1%, 2%, and 3% calcium-hydrolyzed nanoparticles.

The nitrogen adsorption–desorption isotherms and pore-size distributions of the original AAM and AAM samples supplemented with different percentages of calcium-hydrolyzed nano-solutions are depicted in Figure 8. According to IUPAC [89], the original AAM sample showed a nitrogen adsorption–desorption isotherm typical of type IV (Figure 8a). This means that the AAM system exhibited the typical hysteresis loop and had mesopores (2–50 nm in diameter). Otherwise, the AAMs supplemented with calcium-hydrolyzed nano-solutions exhibited isotherm curves conform to type II, which means that the three systems were non-porous or were defined as macroporous systems (>50 nm in diameter).

The BET surface area, average pore diameter, and total pore volume deduced from the nitrogen sorption isotherms of these samples are summarized in Table 4. The specific surface area values decreased in the AAM samples as the content of calcium in the nano-solutions increased, which indicates the densification of the AAM systems at the nanostructure level.

Additionally, it is possible to observe in Figure 8b that the AAM samples supplemented with calcium-hydrolyzed nano-solutions tended to adsorb less nitrogen gas than the original AAM sample as the percentage of calcium-hydrolyzed nano-solutions increased in the AAM systems, indicating that the pore volume of the supplemented AAM systems became smaller with the increase in calcium-hydrolyzed content in the AAM systems, even though the average pore diameter of all the AAM samples was in the range of 3.23–3.44 nm (Table 4). These results indicate that the addition of calcium-hydrolyzed nano-solutions changed the type of porosity and reduced the pore volume of the AAM systems due to the generation of additional binders at the nanometric level, which corroborates the previous results obtained using QXRD, FTIR, and SEM/EDS, where the formation of nanostructured C-S-H, C-A-S-H, and amorphous phase was identified. Therefore, these binders filled pores at the nanostructure level in the AAM systems, making the latter denser and more compact, with a direct compressive strength improvement of the final AAM systems, as the results in Section 3.3.1 indicate.

## 4. Conclusions

The use of non-ionic surfactant (Triton X-100) for the calcium-hydrolyzed nano-solution synthesis process enabled us to produce self-assembled molecular spherical systems (micelles) in aqueous solutions with diameters less than 80 nm that encapsulated the calcium-hydrolyzed nanoparticles with a particle size smaller than 10 nm. The addition of Triton X-100 led to a reduction in the size of the nanoparticles and the formation of reduced amounts of Ca(OH)_2_ nanoparticles that were hexagonal in shape with a homogeneous particle-size distribution. These new well-dispersed nano-solution systems released the smaller nanoparticles into the AAM pastes via the homogeneous distribution of nanoparticles when the nano-solutions were sprayed systematically during the production of these pastes.

The calcium-hydrolyzed nano-solutions were found to have a direct effect on the dissolution of the aluminosilicate phases from the gold MT precursor, increasing the tetrahedral silicate and aluminate monomer concentrations within each sample as the calcium content was increased. In addition, the calcium nanoparticles added to the systems participated in the formation of additional amorphous cementing binders with the presence of low quantities of nanostructured C-S-H and C-A-S-H phases, due to the pozzolanic properties of calcium-hydrolyzed nanoparticles, which allowed them to react with the MT phases and with the hydrated products generated in the MT alkaline activation step.

The AAM specimens with calcium-hydrolyzed nanoparticles showed more consolidated microstructures than the original AAM system. Also, the production of additional amorphous binders due to the reactivity of the MT precursor with added calcium-hydrolyzed nanoparticles improved the final structures at the nanometric level, making them homogeneous, more compact, and denser than the original AAM, which leads to defining AAMs as macroporous systems capable of encapsulating the heavy metals present in MTs. These results suggest that the additional cementing binders generated at micro- and nanostructure levels are responsible for the strength increases of the final AAM specimens, with a direct compressive strength improvement between 44 and 62% in comparison with the original AAM system.

## Figures and Tables

**Table 1:** Theoretical and experimental interplanar distances (d_hkl_).

	Portlandite (Space Group: P-3m1)	
(hkl)	Experimental d_hkl_ (nm)	Theoretical d_hkl_ (nm)
(011)	0.2617	0.2685
(101)	0.2617	0.2685

**Table 2:** QXRD analyses of all AAM specimens. Only phases contributing ≥ 0.2 wt.% of the samples are reported.

Phase	PDF Code	Original AAM wt. %	AAM + 1%Nano wt. %	AAM + 2%Nano wt. %	AAM + 3%Nano wt. %
Quartz	01-077-8621	38.91	37.24	34.76	31.94
Albite	04-017-1022	9.21	8.43	8.13	7.90
Muscovite	00-001-1098	6.32	5.86	4.64	3.10
Portlandite	00-001-1079	-	0.21	0.21	0.20
Calcite	00-024-0027	-	0.21	0.22	0.33
Magnetite	01-075-0449	0.33	0.21	0.21	0.20
Zeolite	01-076-0620	1.20	0.63	0.24	-
C-S-H	01-076-0618	-	0.47	0.57	0.61
C-A-S-H	00-001-1079	0.71	0.83	0.89	0.92
% Amorphous	-	43.07	45.90	50.13	54.72

**Table 3:** Semiquantitative EDS chemical composition of AAM systems.

Element	Semiquantitative Chemical Composition (wt.%)			
	Original AAM	AAM + 1% Nano	AAM + 2% Nano	AAM + 3% Nano
O	36.77	37.89	37.40	39.05
Na	9.23	8.45	8.02	8.05
Mg	0.98	0.83	1.16	1.40
Al	6.76	6.51	6.83	7.53
Si	36.30	37.65	37.12	34.09
K	1.88	1.42	1.56	1.77
Ca	1.48	1.88	2.13	2.37
Fe	6.60	5.37	5.78	5.74

**Table 4:** Comparison of some parameters of the original AAM and AAMs supplemented with calcium-hydrolyzed nano-solutions.

AAM System (Pore Range 2–4 nm)	BET Surface Area (m^2^/g)	Average Pore Diameter (nm)	Total Pore Volume (cm^3/^g) (Pores of 2–4 nm) × 10^−5^
Original AAM	1.151	3.44	5.21
AAM + Nano 1%	1.119	3.31	5.17
AAM + Nano 2%	0.921	3.25	4.14
AAM + Nano 3%	0.918	3.23	4.19

## Data Availability

The data supporting the reported results are not available online.
